# STAT1-Mediated Down-Regulation of Bcl-2 Expression Is Involved in IFN-γ/TNF-α–Induced Apoptosis in NIT-1 Cells

**DOI:** 10.1371/journal.pone.0120921

**Published:** 2015-03-26

**Authors:** Zhao-hui Cao, Quan-you Zheng, Gui-qing Li, Xiao-bo Hu, Shao-long Feng, Gui-lian Xu, Ke-qin Zhang

**Affiliations:** 1 Department of Urology, Institute of Surgery Research, Daping Hospital, Third Military Medical University, Chongqing 400042, China; 2 Department of Immunology, Third Military Medical University, Chongqing 400038, China; 3 Department of Biochemistry and Molecular Biology, School of Pharmacy and Biological Sciences, School of Public Health, University of South China, Hengyang 421001, China; 4 Department of Health Laboratory Technology, School of Public Health, University of South China, Hengyang 421001, China; Emory University, UNITED STATES

## Abstract

Tumor necrosis factor (TNF)-α and interferon (IFN)-γ are the major pro-inflammatory cytokines involved in beta-cell destruction. The fate of islet beta-cells in the cytokine-induced intrinsic mitochondrial apoptotic pathway is determined by the interaction between members of the Bcl-2 family. However, the mechanism through which beta-cell apoptosis is regulated remains unclear. In this study, we treated the murine beta-cell line NIT-1 with TNF-α and IFN-γ and then investigated the regulation of signal transducer and activator of transcription-1 (STAT-1) and expression of the members of the Bcl-2 family in this apoptotic pathway. Results showed that TNF-α and IFN-γ synergistically reduced NIT-1 cell viability. In addition, the decrease in cell growth was due to apoptosis as shown by apoptotic body formation, detected by confocal laser microscope, and a significant increase in Annexin-Vup^+^ cell percentage, detected by flow cytometry. Combination treatment with TNF-α and IFN-γ caused a remarkable increase in the release of cytochrome c, and in the activation of caspase-9 and caspase-3, as well as, an obvious enhancement in STAT-1 phosphorylation; the treatment, however, resulted in the down-regulation in Bcl-2 expression. The enhancement in STAT-1 activity and a down-regulation in Bcl-2 expression was also observed in MIN6 cells, another murine beta-cell derived line, after cells exposure to the combination of TNF-α and IFN-γ treatment. Knockdown of STAT-1 gene expression by siRNA or inhibition of STAT-1 activation with fludarabine reversed Bcl-2 down-expression and led to a significant decrease in apoptosis in TNF-α- and IFN-γ-treated NIT-1 cells. Taken together, our results suggest that STAT1-mediated down-regulation of Bcl-2 is involved in NIT-1 cell apoptosis induced by combination treatment with TNF-α and IFN-γ.

## Introduction

Type-1 diabetes mellitus (T1DM) is an autoimmune disease characterized by chronic inflammation and selective destruction of insulin-producing beta-cells. Pancreatic beta-cell death is primarily caused by apoptosis [1–4]. Many factors induce beta-cell apoptosis, including the invasion of autoreactive T lymphocytes and macrophages into the islets of Langerhans. Pro-inflammatory cytokines such as interferon (IFN)-γ, tumor necrosis factor (TNF)-α, and interleukin (IL)-1β released by infiltrative T lymphocytes and macrophages, together with FasL, perforin, and granzyme B, are considered the main factors leading to beta-cell apoptosis. Moreover, beta-cell apoptosis is induced by various kinds of cytokine combinations, but not by a single cytokine. The combination and distribution of cytokines are different in different animal models [5–7]. Further understanding of the apoptotic pathways activated by different cytokine combinations in beta-cells is necessary to develop individualized therapies to prevent beta-cell destruction in T1DM.

The classical apoptotic pathways include endoplasmic reticulum stress pathways, extrinsic death receptor pathways, and intrinsic mitochondrial pathways. In the mitochondrial pathway, mitochondria play a key role in triggering cell death. Transcriptional and post-transcriptional modification and protein-protein interactions between members of Bcl-2 family determine the fate of the cells in this pathway [8, 9]. When combined with TNF-α, IFN-γ secreted by activated T lymphocytes is involved in beta-cell apoptosis via the mitochondrial pathway [10]. Activation of signal transducer and activator of transcription-1 (STAT-1) was implicated in IFN-γ- and TNF-α- induced beta-cell apoptosis [11, 12]. Our previous work demonstrated that Nuclear factor-kappa B (NF-κB)-mediated down-regulation of Bcl-2 is involved in mediating IFN-γ- and TNF-α- induced caspase-3 activation in the cell line MIN6 [13]. However, it is not clear whether the expression of the members of the Bcl-2 family is regulated by STAT-1.

In the present study, we used the mouse pancreatic beta-cell line NIT-1 [14–16] to investigate the role of the major proteins in the Bcl-2 family in IFN-γ- and TNF-α- induced beta-cell apoptosis and to study the relationship between the expression of Bcl-2 proteins and STAT-1 activation.

## Materials and Methods

### Cell culture and treatment

NIT-1 cells (Hanbo Company of Biotechnology, Shanghai, China) and MIN6 cells (Kindly provided by Dr. Fen Zhang, Department of Endocrine, Tongren Hospital, Beijing, China) were grown in DMEM culture medium containing 25 mM glucose (Invitrogen) supplemented with 15% FBS, 100 μg/mL streptomycin, 100 U/mL penicillin, and 2 mM glutamine.

### MTT assays

NIT-1 cells were cultured in 96-well culture plates at an initial density of 2 × 10^4^ cells/well and treated with either 100 ng/mL IFN-γ, 10 ng/mL TNF-α, or a combination of both IFN- γ and TNF-α for 48 h. In some experiments, the cells were seeded at an initial density of 1 × 10^4^ cells/well and treated with 100 ng/mL IFN-γ, 10 ng/mL TNF-α, or a combination of both IFN-γ and TNF-α for 0, 24, 48 and 72 h. In some cases, the cells were pretreated with 100 μM of the STAT-1 inhibitor, fludarabine (Sigma), for 1 h before IFN-γ and TNF-α treatment. After cytokine treatment, cell viability was determined by the MTT assay (Sigma) as previously described [13].

### Morphological assessment of apoptotic cells

NIT-1 cells were seeded in 96-well culture plates at 1 × 10^4^ cells/well and exposed to 10 ng/mL TNF-α plus 100 ng/mL IFN-γ for 48 h. Cell morphology was observed under the microscope (Olympus 1X71S8F-2, Japan). In some experiments, cells were seeded onto coverslips at 3 × 10^5^ cells/well in 6-well culture plates and treated with 100 ng/mL IFN-γ plus 10 ng/mL TNF-α for 48 h. Cells were harvested, washed with PBS, stained with 5 μg/mL of Hoechst 33258 dye (Sigma), and incubated in the dark at room temperature for 20 min. Chromatin condensation of the cells was observed under the confocal laser scanning microscope (Leica microsystem), and images were obtained.

### Flow cytometry

NIT-1 cells were treated with IFN-γ and TNF-α as described above for 48 h. In some cases, cells were pretreated with 100 μM fludarabine (Sigma), a STAT-1 inhibitor, for 1 h before IFN-γ (100 ng/mL) and TNF-α (10 ng/mL) treatment. Cells were harvested and double stained with Annexin-V-FITC Apoptosis Detection Kit (eBioscience) and 7-AAD (eBioscience) according to the manufacturer’s suggested protocols. The percentage of apoptotic cells was analyzed by flow cytometry (BD, FACS Canto II).

In some experiment, cells were transfected with 60 nM of STAT-1 siRNA (Santa Cruz) or control siRNA (Santa Cruz), according to the manufacturer’s suggested siRNA transfection protocol and than treated with IFN-γ (100 ng/mL) and TNF-α (10 ng/mL) for 1 h. Cells were collected and stained with mouse anti-Stat1 antibody (Abcam), followed by DyLight^TM^ 488 goat anti-mouse IgG (Biolegend). The percentage of STAT-1 was analyzed by flow cytometry (BD, FACS Canto II).

### Western blot

NIT-1 cells were seeded at 3 × 10^5^ cells/well in 6-well plates and treated with IFN-γ or TNF-α alone or a combination of IFN-γ and TNF-α for indicated times. In some experiments, cells were pretreated with 100 μM STAT1 inhibitor, fludarabine, for 1 h and then treated with IFN-γ and TNF-α for indicated times. At the end of the culture time, both the floating cells and attached cells were harvested. Monoclonal antibodies against cleaved caspase-3 (Cell Signaling), caspase-3 (Cell Signaling), caspase-9 (Beyotime Institute of Biotechnology), Stat-1 (Cell Signaling), phospho-Stat1 (Tyr701) (Cell Signaling), Stat-3 (Cell Signaling), Phospho-Stat3 (Cell Signaling), Bcl-2 (Abcam), Bax (Beyotime Institute of Biotechnology), cytochrome c (Cell Signaling), and COX4 (BD Biosciences) were used to analyze the expression of the respective proteins by western blotting as previously described [13]. Protein levels were calculated relative to that of β-actin (Beyotime Institute of Biotechnology).

### Quantitative PCR

NIT-1 cells were seeded at 4 × 10^5^ cells/well in 6-well plates and treated with a combination of IFN-γ and TNF-α for indicated times. Total RNA was extracted using Trizol (Roche) and then reverse transcribed into cDNA by using RT kit (TOYOBO). Quantitative PCR with SYBR Green Premix DimerEraser (Takara) was performed using an ABI 7000 sequence detection system and mRNA levels were normalized according to levels of the housekeeping gene GAPDH. Primer sequences for both quantitative PCR amplification were as follows: Bcl-2 forward, 5′-GTACCTGAACCGGCATCTG-3'; Bcl-2 reverse, 5′-GGGGCCATATAGTTCCACAA-3′. GAPDH forward, 5′-ACCACAGTCCATGCCATCAC-3′; GAPDH reverse, 5′-TCCACCACCCTGTTGCTGTA-3′.

### Immunofluorescence staining

NIT-1 or MIN6 Cells (2×10^5^ cells/well) were seeded on coverslips of plat-bottom 6-well microtiter plates and treated with IFN-γ (100 ng/ml) and TNF-α (10 ng/ml) in combination for indicated times. In some experiment, cells were transfected with 60 nM of STAT-1 siRNAs (Santa Cruz) or control siRNA (Santa Cruz) as described above, before IFN-γ and TNF-α treatment. Cells were stained with rabbit anti-Bcl-2 antibody (Abcam) or mouse anti-Stat1 (phospho Y701) antibody and then with DyLight^TM^ 488 donkey anti-rabbit IgG (Biolegend) and DyLight^TM^ 488 goat anti-mouse IgG (Biolegend), respectively, followed by 5μg/ml of Hoechst 33258 (Sigma) staining. Cells were imaged under a confocal laser scanning microscope (Leica microsystem).

### Statistical analysis

Results are shown as the mean ± standard deviation unless otherwise stated. GraphPad Prism 5.0 (GraphPad Software, Inc.) was used for statistical analysis. Statistical analysis was performed using unpaired t test, and *p* < 0.05 was considered statistically significant.

## Results

### Combination treatment with IFN-γ and TNF-α promotes NIT-1 cell apoptosis

**Fig 1:**
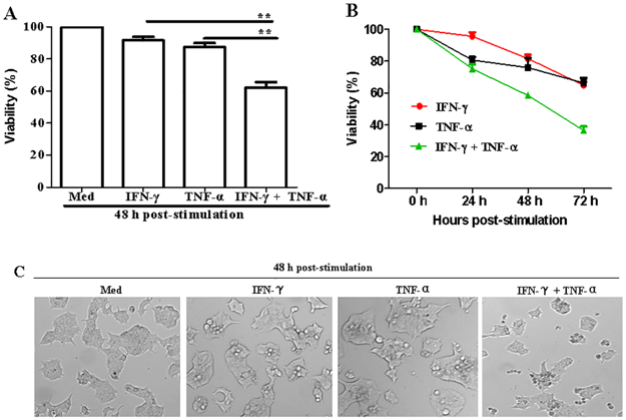
The effect of IFN-γ and TNF-α combination treatment on NIT-1 cell viability. (A) Cells (2 × 10^4^/well) were seeded in 96-well microtiter plates and stimulated with IFN-γ (100 ng/mL) or TNF-α (10 ng/mL) alone or in combination for 48 h. (B) Cells (1 × 10^4^/well) were stimulated with IFN-γ (100 ng/mL) or TNF-α (10 ng/mL) alone or in combinations for 0, 24, 48, and 72 h. Cell viability was measured by the MTT assay. The OD value at 490 nm of cells cultured in medium was set to 100%. Data are presented as mean ± SEM. (C) Morphological changes of NIT-1 cells induced by IFN-γ/TNF-α combination treatment. Cells (2 × 10^4^/well) were seeded in 96-well microtiter plates and stimulated with IFN-γ or TNF-α alone, or in combination for 48 h. The cells were then observed using the phase contrast microscope. Magnification, ×100. Data represent three independent experiments. **p ≤ 0.01.

IFN-γ and TNF-α are the two key pro-inflammatory cytokines responsible for the destruction of islet cells *in vitro* [17]. In order to identify the underlying mechanism, NIT-1 cells were treated with TNF-α, IFN-γ, or a combination of TNF-α and IFN-γ. As shown in Fig. 1A, the combination of IFN-γ and TNF-α significantly reduced cell viability (IFN-γ & TNF-α + IFN-γ: *p* = 0.0019; TNF-α & TNF-α + IFN-γ: *p* = 0.0040). Similarly, when the cells were treated with cytokines, cell viability decreased with increasing treatment duration (Fig. 1B). These results indicate that IFN-γ and TNF-α significantly inhibit NIT-1 cell growth. It was also shown that this observed cell growth inhibition was due to cell death, which was indicated by the change in the shape of the cells from adhering to round and floating after IFN-γ and TNF-α treatment for 48 h (Fig. 1C).

**Fig 2:**
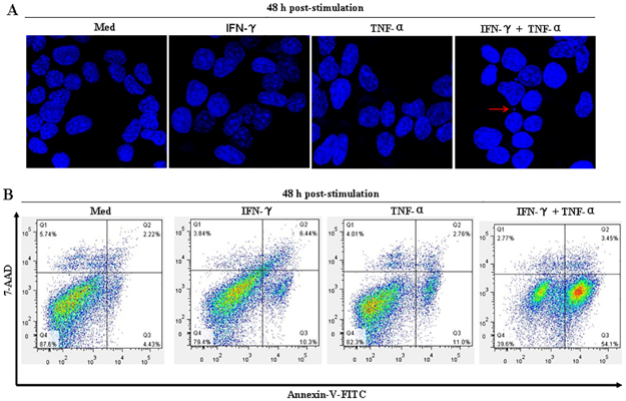
IFN-γ and TNF-α synergistically induced NIT-1 cell apoptosis. (A) Nuclear changes in the cells were detected by staining the cells with Hoechst 33258, a DNA-binding fluorochrome. Cells (3 × 10^5^/well) were seeded on coverslips in 6-well plates and treated with IFN-γ (100 ng/mL) or TNF-α (10 ng/mL) alone, or in combination for 48 h. Cells were collected and stained with Hoechst 33258 solution (5 μg/mL). Nuclei were observed with a confocal laser scanning microscope. The red arrow indicates a representative apoptotic body. (B) Cells (1 × 10^5^/well) were treated with IFN-γ (100 ng/mL) or TNF-α (10 ng/mL) alone, or in combination for 48 h. Cells were harvested and double-stained with Annexin V and 7-AAD. Apoptotic cell (Annexin V^+^ and 7-AAD^-^ cells) percentage was determined by flow cytometry. Data are representative of three independent experiments.

Since cell death includes necrosis, apoptosis, and autophagic cell death, Hoechst 33258 staining was performed to investigate which form of cell death was induced by IFN-γ and TNF-α in the NIT-1 cells. Results showed that cells treated with IFN-γ and TNF-α undergo an obvious apoptotic morphological change, including chromatin condensation and the formation of apoptotic bodies (Fig. 2A).

Cells in early apoptosis usually expose the phosphatidylserine to the outer leaflet of the plasma membrane. According to their Annexin-V affinity, apoptotic cells (Annexin-V^+^) could be distinguished from living cells (Annexin-V^-^) by flow cytometry [13]. Furthermore, the double-labeling assay (Annexin-V combined with 7-AAD) could distinguish necrotic or late apoptotic cells (Annexin-V^+^/7-AAD^+^) and early apoptotic cells (Annexin-V^+^/7-AAD^-^) [18]. As seen in Fig. 2B, after treatment with a combination of IFN-γ and TNF-α for 48 h, the percentage of early apoptotic cells increased to 54.1%, but the percentage of necrotic or late apoptotic cells remained less than 5.0%. These results further demonstrated that NIT-1 cell death induced by a combination treatment with IFN-γ and TNF-α is due to apoptosis rather than necrosis.

### Combination treatment with IFN-γ and TNF-α induced mitochondrial stress in NIT-1 cells

**Fig 3:**
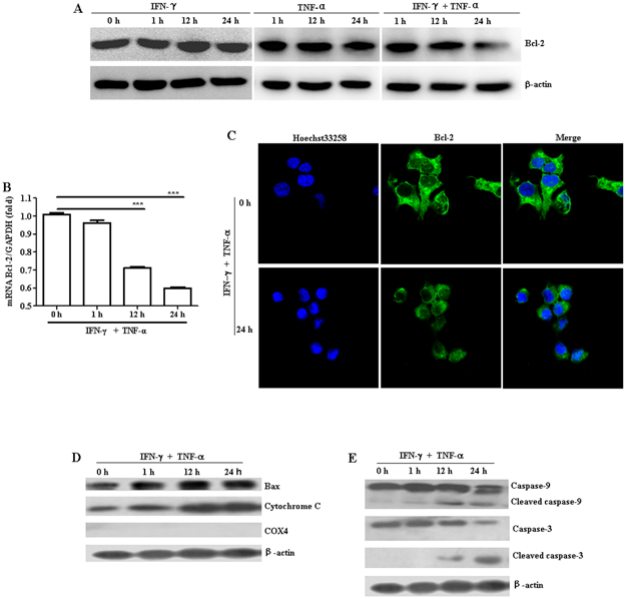
The mitochondrial pathway was involved in IFN-γ/TNF-α–induced NIT-1 cell apoptosis. NIT-1 cells were treated with IFN-γ (100 ng/mL) or TNF-α (10 ng/mL) alone or in combination for indicated times. (A) The expression of Bcl-2 was evaluated by western blot. (B) The level of Bcl-2 mRNA was determined by quantitative PCR. mRNA levels were normalized according to levels of the housekeeping gene GAPDH. ***p ≤ 0.001. (C) The expression of Bcl-2 was observed by the confocal laser scanning microscope. Nuclei were stained with Hoechst 33258 solution (5 μg/mL). (D) Bax expression and cytochrome c release was evaluated by western blot. (E) The expression of cleaved caspase-3 and caspase-9 was evaluated by western blot. COX4, which is located exclusively in the mitochondria, was used here to confirm whether the cytoplasmic protein fractions include mitochondrial proteins. Equal protein loading in all lanes was confirmed by probing the blots with anti-β-actin antibody. Data shown are representative of two independent experiments.

To investigate whether combination treatment with TNF-α and IFN-γ induces mitochondrial stress in NIT-1 cells, we determined the effects of TNF-α and IFN-γ exposure on Bcl-2 and Bax expression, and on mitochondrial cytochrome c release at the indicated times. Although IFN-γ or TNF-α treatment alone didn’t show obvious effects on Bcl-2 levels (Fig. 3A), it was found that Bcl-2 expression levels of both protein and mRNA was obviously down-regulated (Fig. 3A-C) while Bax expression and cytochrome c release was remarkably increased (Fig. 3D) over time after the combination treatment with TNF-α and IFN-γ. The results suggest that TNF-α and IFN-γ treatment enhanced cytochrome c release, which may be related to a decrease in the proportionality of Bcl-2 and Bax expression, in turn changed the structure of the mitochondrial membranes.

Caspase-3 and caspase-9 play crucial roles in the apoptotic mitochondrial pathway [19, 20]. To examine if caspase-3 and caspase-9 activation is involved in the TNF-α- and IFN-γ–mediated apoptosis of NIT-1 cells, the expression of the cleaved caspase-3 and caspase-9 was analyzed by western blot. As illustrated in Fig. 3E, the combination of TNF-α and IFN-γ caused a time-dependent cleavage of caspase-3 and caspase-9.

### TNF-α promoted IFN-γ-mediated STAT-1 activation in NIT-1 cells

**Fig 4:**
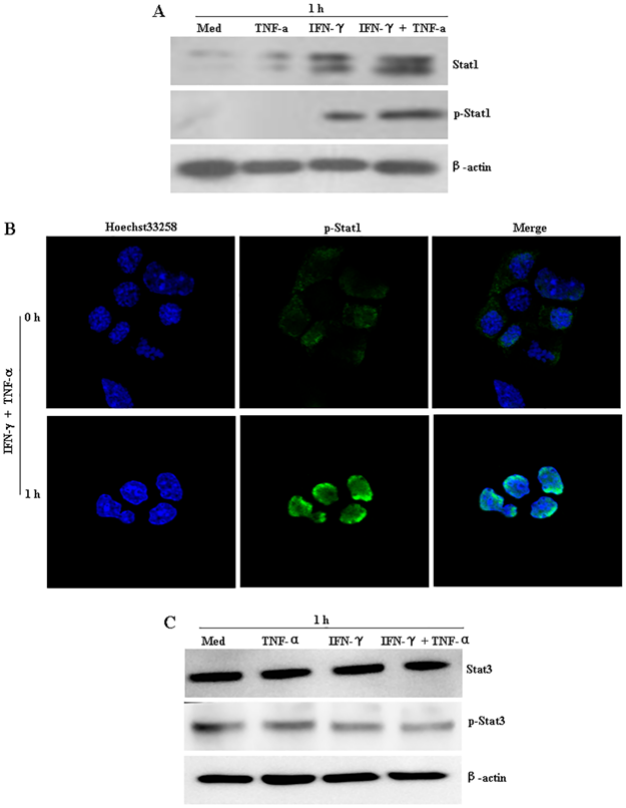
IFN-γ/TNF-α-mediated STAT-1 activation in NIT-1 cells. NIT-1 cells were treated with IFN-γ (100 ng/mL) or TNF-α (10 ng/mL) alone or in combination for 1 h. (A) The expression of STAT-1 and phospho- STAT1 was then determined by western blot. (B) The nuclear localization of phosphorylated STAT-1 was then observed by the confocal laser scanning microscope. Nuclei were stained with Hoechst 33258 (5 μg/mL). (C) The expression of STAT-3 and phospho- STAT3 was then determined by western blot. Equal protein loading in all lanes was confirmed by probing the blots with anti-β-actin antibody. Data are representative of three independent experiments.

**Fig 5:**
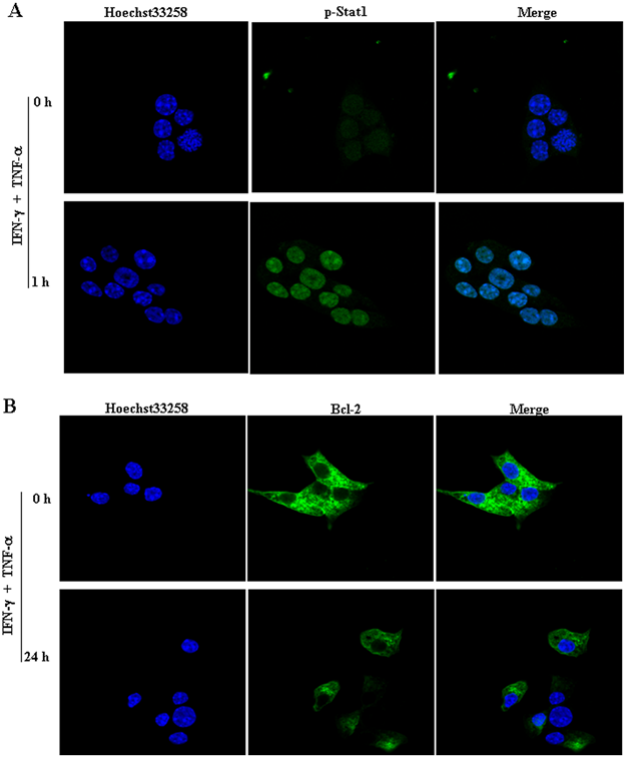
IFN-γ/TNF-α-mediated STAT-1 activation and down-regulation of Bcl-2 expression in MIN6 cells. Cells were treated with the combination of IFN-γ (100 ng/mL) and TNF-α (10 ng/mL) for indicated times. The expression of phospho- STAT1 (A) and Bcl-2 (B) was then determined by the confocal laser scanning microscope. Nuclei were stained with Hoechst 33258 solution (5 μg/mL). Data are representative of two independent experiments.

To further explore the upstream events that occurred during mitochondrial stress induced by the combination of IFN-γ and TNF-α, we considered the possible involvement of STAT-1, an important component in IFN-γ signal transduction [21]. Results showed that, although TNF-α alone did not induce STAT-1 phosphorylation, cells treated with the IFN-γ and TNF-α combination had obviously higher levels of both total STAT-1 and phosphorylated STAT-1 than did the untreated controls or cells treated with IFN-γ or TNF-α alone (Fig. 4A), indicating that TNF-α enhanced IFNγ-mediated STAT-1 activation in NIT-1 cells. Moreover, an obvious nuclear localization of phosphorylated STAT-1 was also observed after cells exposure to IFN-γ and TNF-α treatment for 1 h (Fig. 4B). However, the regulation of signal transducer and activator of transcription-1 (STAT-3) was constitutively active and presented a slight down-regulation after the combination of IFN-γ and TNF-α treatment (Fig. 4C), which is consistent with others results in the cancer cells [22]. Notably, an enhancement in STAT-1 activity and the down-regulation in Bcl-2 expression were also observed in IFN-γ/TNF-α-treated MIN6 cells, another murine beta-cell derived line (Fig. 5A and B).

### NIT-1 cell apoptosis induced by combination treatment with IFN-γ and TNF-α was regulated by STAT1-mediated down-regulation of Bcl-2 expression

**Fig 6:**
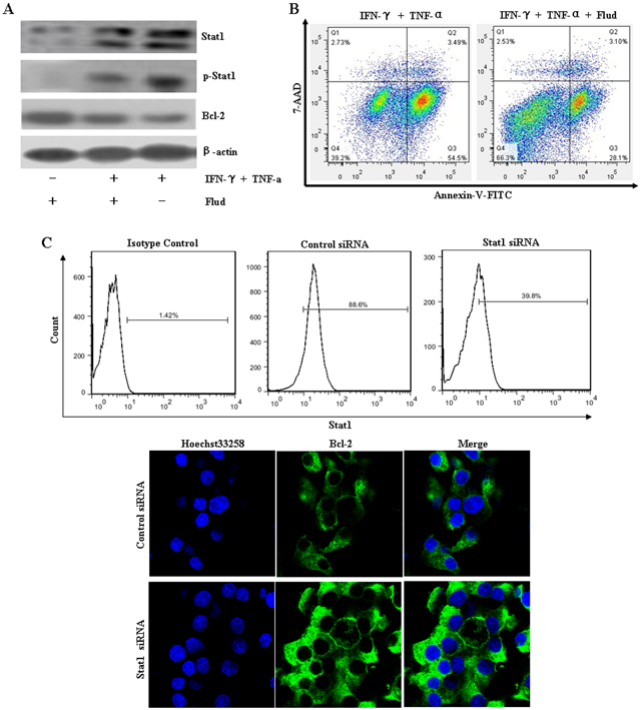
IFN-γ/TNF-α-induced NIT-1 cell apoptosis was regulated by STAT1-mediated down-regulation of Bcl-2 expression. (A) After being pretreated with or without STAT1 inhibitor, fludarabine (100 μM), for 1 h, the cells were treated with a combination of IFN-γ and TNF-α for 1 h or 24 h. The expression of STAT-1 (at 1 h), phospho- STAT1 (at 1 h), and Bcl-2 (at 24 h) was determined by western blot. Equal protein loading in all lanes was confirmed by probing the blots with anti-β-actin antibody. (B) Cells pretreated with fludarabine (100 μM) for 1 h were stimulated with IFN-γ and TNF-α combination for 48 h. Cells were harvested and double-stained with Annexin V and 7-AAD. The percentage of apoptotic cells was determined by flow cytometry. (C) After being transfected with Stat1 siRNA or control siRNA, according to the manufacturer’s suggested siRNA transfection protocol, cells were treated with IFN-γ (100 ng/mL) and TNF-α (10 ng/mL) in combination for 1 h or 24 h. The expression of STAT-1 (at 1 h) was determined by flow cytometry. Bcl-2 (at 24 h) expression was observed by the confocal laser scanning microscope. Nuclei were stained with Hoechst 33258 solution (5 μg/mL). Data shown are representative of two independent experiments.

Fludarabine is a specific inhibitor of STAT-1 signaling [23–25]. To further investigate the regulation role of STAT-1 in Bcl-2 expression, cells were pretreated with fludarabine for 1 h and then stimulated with IFN-γ and TNF-α. Results showed that fludarabine treatment reversed cytokine-induced Bcl-2 down-regulation (Fig. 6A), and markedly decreased the percentage of apoptotic cells (Fig. 6B). Similar results were obtained when STAT-1 gene expression was knockdown in NIT-1 cells by siRNA (Fig. 6C). All these results suggest that the STAT-1/Bcl-2 pathway is involved in IFN-γ and TNF-α-mediated NIT-1 cell apoptosis.

## Discussion

TNF-α and IFN-γ are the most likely cytokines to be acting in synergy during the inflammation of pancreatic beta-cells. TNF-α and IFN-γ treatment can lead to the beta-cell destruction via the induction of the mitochondrial apoptosis pathway involving the transcription factor STAT-1 [10, 26]. However, the underlying mechanism by which STAT-1 regulates its downstream molecules to mediate beta-cell death is still not understood. In this study, we investigated the cytokine-induced intrinsic mitochondrial apoptotic pathway in the murine pancreatic beta-cell line NIT-1.

Our results showed that the combination of IFN-γ and TNF-α could induce apoptosis, which was in agreement with the results obtained from a previous study [27]. Caspase-3, activated by the initiator caspase-9, is an executive caspase, which receives the apoptosis signal from the mitochondria; the signal is then transmitted to Poly (ADP-ribose) polymerase (PARP), which is associated with the cleavage of genomic DNA [28]. In this study, we found that, after the cells were treated with a combination of IFN-γ and TNF-α, cytochrome c release was increased and caspase-9 and caspase-3 were activated. These results indicated that TNF-α and IFN-γ induced apoptosis through mitochondrial dysfunction and cytochrome c release.

The intrinsic mitochondrial apoptotic pathway is tightly controlled by pro- and anti-apoptotic members of the Bcl-2 family [8, 9]. Bcl-2 is an anti-apoptotic protein of the Bcl-2 family, which can block the activation of the caspase cascade initiated by mitochondrial release of cytochrome c. Bax is a pro-apoptotic protein of the Bcl-2 family, which can induce mitochondrial permeabilization [29]. However, it is still unclear whether cytokine-induced cell death is dependent on Bcl-2 family proteins. It was previously reported that Bcl-2 was not significantly influenced by TNF-α and IFN-γ treatment of MIN6 insulinoma cells [17]. IL-1β—and IFN-γ-induced beta-cell death was also independent of Bax in INS-1 cells and rat islets [30]. In contrast, Grunnet et al. observed an effect of Bax activity on cytokine-induced beta-cell apoptosis in the canonical mitochondrial pathway in INS-1 cells and human islets [6]. Consistent with the results of the study by Grunnet et al., we found that after NIT-1 cells were exposed to TNF-α and IFN-γ-treatment, Bcl-2 expression was gradually down-regulated, while Bax expression was remarkably up-regulated. The discrepancy among these results may be related to the level of expression of pro-inflammatory cytokines during insulitis; alternatively, Bcl-2 proteins could trigger beta-cell apoptosis under some conditions [9].

STAT-1 is a key member of the IFN-γ–mediated signal pathway, which is important for cytokine-induced beta-cell apoptosis [31]. Activation of STAT-1 also promotes cell death [32, 33]. We confirmed that STAT1 activation was involved in IFN-γ—and TNF-α-induced NIT-1 cell death, but it is not clear whether STAT-1 activation is associated with the expression of Bcl-2 or Bax. In this study, we found an obvious reduction in the expression of the anti-apoptosis protein Bcl-2 and a significant increase in the expression of the pro-apoptotic protein Bax after as early as 12 h of TNF-α and IFN-γ treatment. Pretreatment of NIT-1 cells with the STAT-1 inhibitor, fludarabine, reversed Bcl-2 down-regulation and Bax up-regulation, and increased cell viability significantly. This indicated that STAT1-mediated down-regulation of Bcl-2 and up-regulation of Bax is involved in NIT-1 cell apoptosis induced by the combination treatment with IFN-γ and TNF-α.

In conclusion, our results clearly show that IFN-γ and TNF-α induced NIT-1 cell apoptosis via the mitochondrial pathway and that this induction is brought about through STAT1-mediated regulation of Bcl-2/Bax expression.
